# SOCIETAL PERSPECTIVE ON COST DRIVERS FOR HEALTH TECHNOLOGY ASSESSMENT IN SINDH, PAKISTAN

**DOI:** 10.1017/S0266462317000320

**Published:** 2017

**Authors:** Asif Raza Khowaja, Craig Mitton, Rahat Qureshi, Stirling Bryan, Laura A. Magee, Peter von Dadelszen, Zulfiqar A. Bhutta

**Affiliations:** 1 Department of Obstetrics and Gynaecology, and British Columbia Children's Hospital, University of British Columbia, Centre for Clinical Epidemiology and Evaluation, Vancouver Coastal Health Research Institute, Division of Women & Child Health, Aga Khan University; 2 Centre for Clinical Epidemiology and Evaluation, Vancouver Coastal Health Research Institute, School of Population and Public Health, University of British Columbia, craig.mitton@ubc.ca; 3 Division of Women & Child Health, Aga Khan University; 4 Centre for Clinical Epidemiology and Evaluation, Vancouver Coastal Health Research Institute, School of Population and Public Health, University of British Columbia; 5 Molecular and Clinical Sciences Research Institute, St George's, University of London, Department of Obstetrics and Gynaecology, St George's University Hospitals NHS Foundation Trust; 6 Division of Women & Child Health, Aga Khan University, Program for Global Pediatric Research, Hospital For Sick Children, Toronto

**Keywords:** Health technology assessment, Cost drivers, Economic model, Maternal, newborn, and child health (MNCH), Low- and middle-income-countries (LMICs)

## Abstract

**Background:** Understanding cost-drivers and estimating societal costs are important challenges for economic evaluation of health technologies in low- and middle-income countries (LMICs). This study assessed community experiences of health resource usage and perceived cost-drivers from a societal perspective to inform the design of an economic model for the Community Level Interventions for Pre-eclampsia (CLIP) trials.

**Methods:** Qualitative research was undertaken alongside the CLIP trial in two districts of Sindh province, Pakistan. Nine focus groups were conducted with a wide range of stakeholders, including pregnant women, mothers-in-law, husbands, fathers-in-law, healthcare providers at community and health facility-levels, and health decision/policy makers at district-level. The societal perspective included out-of-pocket (OOP), health system, and program implementation costs related to CLIP. Thematic analysis was performed using NVivo software.

**Results:** Most pregnant women and male decision makers reported a large burden of OOP costs for in- and out-patient care, informal care from traditional healers, self-medication, childbirth, newborn care, transport to health facility, and missed wages by caretakers. Many healthcare providers identified health system costs associated with human resources for hypertension risk assessment, transport, and communication about patient referrals. Health decision/policy makers recognized program implementation costs (such as the mobile health infrastructure, staff training, and monitoring/supervision) as major investments for the health system.

**Conclusions:** Our investigation of care-seeking practices revealed financial implications for families of pregnant women, and program implementation costs for the health system. The societal perspective provided comprehensive knowledge of cost drivers to guide an economic appraisal of the CLIP trial in Sindh, Pakistan.

Globally, maternal and newborn mortality trends have declined, albeit slowly, over the past 10 years in low- and middle-income countries (LMICs) (1). Rapidly increasing costs of care, shortages of trained personnel, cultural barriers that delay care-seeking, and geographical remoteness continue to impose considerable challenges for health systems in LMICs that are struggling to achieve new sustainable development goals for mortality reduction by 2030 (2). In response, policy makers are considering technological innovations such as mobile health (mHealth) interfaces to bridge gaps in maternal, newborn, and child health (MNCH) service coverage in LMICs. Currently, health technologies are used for early detection of disease, vaccination reminders, behavior change for child survival interventions, and training/retention of healthcare providers (3).

Economic evaluation, conducted as part of health technology assessment (HTA), provides a systematic approach to collect evidence about costs and effectiveness from the diverse perspectives of care providers, care receivers, and community stakeholders (4). Such an inclusive approach is imperative to guide the use of health technologies and inform policy decisions on resource allocation for program scale-up. The literature on economic evaluation embedded in HTA is sparse; of the 1,412 studies currently registered with clinical trials, only 124 (~9 percent) are in LMICs (5). A knowledge gap in this area undermines future investment for technological innovations in health care in LMICs.

Pakistan is the world's sixth largest country in terms of population and belongs to a group of LMICs according to the World Bank classification (i.e., gross national income per capita in international dollar $1,046 to $4,125) (6). Pakistan's healthcare delivery system is bifurcated into private and public sectors. The private health sector includes informal care providers (i.e., traditional birth attendants, spiritual healers, and quack doctors) and formal medical clinics and/or hospitals. The public health sector is comprised of primary, secondary, and tertiary levels of health facilities (7). An extension of primary level care includes community-based Lady Health Workers (LHWs) recruited by the National Program for Family Planning and Primary Healthcare. LHWs are aged 18 to 45 years and are local residents who have attended a minimum 8 years of schooling. Having received 15 months of on-the job training in the area of MNCH, a LHW covers a catchment of 1,000 people and provides door-to-door basic services inclusive of antenatal care (ANC), vaccination, health education, and psychological counseling on reproductive health and family planning (8).

Overall, healthcare-seeking practices portray a dismal picture suggesting that only 65 percent of pregnant women seek routine ANC; nearly 48 percent of deliveries occur without the assistance of a skilled care provider and fewer than 50 percent of women seek either postpartum and/or newborn care (9). It is estimated that the maternal mortality ratio is as high as 276 per 100,000 live births; and infant mortality rate is as high as seventy-four per 1,000 live births (9).

The Community-Level Interventions for Pre-eclampsia (CLIP) cluster randomized controlled trial (cRCT) is testing an innovative package of care that introduces mHealth platform-guided case identification, time-of-disease risk stratification, and case management for women with a hypertensive disorder of pregnancy (HDP) in Pakistan, India, and Mozambique (10). The assessment of cost-effectiveness of the CLIP trial remains a priority research question endorsed by implementing partners and the Ministry of Health. A literature search yielded a small number of cost-effectiveness studies in LMICs (11), and the existing studies were of limited help in designing an economic model. Previous economic studies on pre-eclampsia diagnosis and case management restricted focus to health provider costs, such as the cost of drugs, devices, and human resource (12).

The societal perspective, inclusive of out-of-pocket (OOP) costs to patients/families and opportunity costs, albeit a very important driver for public policy, were excluded from these cost-effectiveness analyses. To design an economic model suitable for the needs of stakeholders evaluating the CLIP interventions in Pakistan, this study aimed to assess community experiences of health resource usage and perceived cost drivers, using a societal perspective.

## METHODS

### Study Design and Conceptual Framework

**Figure 1.**
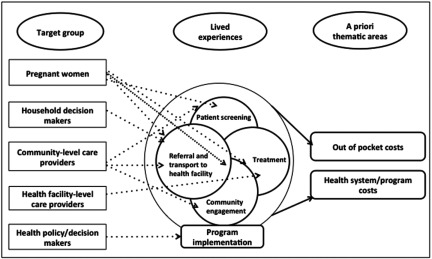
Conceptual framework and approach for societal perspective.

Qualitative research was undertaken alongside the CLIP trial during November to December 2014. The conceptual framework was guided by a phenomenological approach that attempts to understand people's experience in regard to specific phenomenon of common interest. It is based on participants’ view of the situation and how they interpret those experiences (13). In relevance to our study, we explored lived experiences about costs of patient screening, referral, treatment, and transport to health facilities in a diverse group of participants (Figure 1).

### Study Setting

This study was conducted in two districts (i.e., Matiari, and Hyderabad) of the southern Sindh province in Pakistan. Matiari is a rural district located 25 kilometers north of Hyderabad with a population of approximately 0.6 million (14). Hyderabad is a semi-urban setting with a population of over 1 million, making it the second largest district of Sindh province (15). A vast majority of residents are Muslims, agriculturalist by occupation, and Sindhi/Urdu languages are widely spoken in these districts. Overall, literacy rates are 40 percent in Matiari and 50 percent in Hyderabad.

### Participant Inclusions/Exclusions

Beyond the published CLIP trial protocol (10), pregnant women were eligible for this qualitative study if they were: (i) within an intervention cluster; (ii) identified as being hypertensive (i.e., systolic blood pressure > 140 mm Hg) during a CLIP pilot cRCT visit; and (iii) available for a discussion lasting at least 60 minutes. The literature suggested that household decision makers traditionally make decisions about women's health issues in LMICs (16). As inclusively as possible, household decision makers (e.g., husbands, mothers-in-law, fathers-in-law) of hypertensive pregnant women were eligible if they also expressed availability for at least 60 minutes of discussion. LHWs, and medical doctors (MDs) were eligible, if they participated in the implementation of the CLIP pilot cRCT. The health decision/policy makers were eligible if they were involved in decisions related to the execution of the CLIP pilot cRCT. Participants’ characteristics varied in terms of age, schooling years, and occupation (Table 1).

**Table 1.** Focus Group Participants’ Characteristics

	No. of focus	No. of	Age in years	Schooling years	Occupation (*n*, frequency
Groups	groups	participants	Mean (+SD)	Mean (+SD)	of participants)
Pregnant women with pregnancy hypertension	02	12	24.5 (3.8)	3.1 (4.7)	8 Housewife 4 unskilled labour work
Mothers-in-law		07	44.2 (4.2)	0 (-)	6 Housewife 1 unskilled labour work
Husband of pregnant women with pregnancy hypertension	02	13	26.3 (5.2)	3.7 (4.1)	9 unskilled labour work 3 Farmer 1 Employed in armed services
Fathers-in-law		04	43.8 (2.1)	0.75 (1.5)	2 Farmer; 1 unskilled labour work 1 Business
Community care providers	02	19	31.2 (4.8)	11.1 (2.0)	19 Employed as LHW
Medical doctors at referral health facilities	02	20	33.7 (5.6)	17.8 (1.1)	11 OBGYN specialist 9 General physician
District health decision/policy makers	01	10	53.4 (3.3)	17.1 (1.2)	8 General physician 1 Specialists 1 Administration
Total	09	85			

OBGYN, Obstetrics and gynecologist.

### Sampling Procedures

A purposive sampling strategy was imperative to this study design as we needed individuals or groups with lived experiences (i.e., information-rich cases) who could provide insight based on their own experiences related to HDP care sought/provided during the CLIP pilot cRCT. The sampling framework included four intervention clusters (~400 pregnancies per cluster in one year), in which we estimated 80 pregnant women would experience HDP (i.e., 5 percent of all pregnancies). Pregnant women and household decision makers were identified through the trial recruitment logs, while healthcare providers and decision/policy makers were identified through health facility networks.

The project-trained research assistants (RAs) approached eligible participants in their respective settings (i.e., either at their home or workplace) and invited them to take part in the study. We had anticipated five focus groups (FGs) (at least one FG per each group); however, we increased the number of FGs (two FGs per group; except health decision/policy makers) to achieve data saturation. In total, nine FGs were conducted: two with pregnant women/mothers-in-law (*n* = 19 in total), two with husbands/fathers-in-law (*n* = 17), two with LHWs (*n* = 19), two with medical doctors at health facilities (*n* = 20), and one with district-level health decision/policy makers (*n* = 10). Participants were reimbursed for study-related transportation costs.

### Data Collection

The FG guides were developed from the literature review. Key constructs included OOP costs related to care-seeking during pregnancy (17) and health service usage in the context of LMICs (18). Participants of the study were asked to reflect on lived experiences and interpret situations related to financial costs as a result of their participation in a 1-year pilot phase of the CLIP cRCT. FG guides were developed in English as the main language of literature review. FG guides were translated into Sindhi; and back translated into English. They were pilot-tested for comprehension and cultural sensitivity. Native Sindhi-speaking and project-trained RAs moderated the FGs. The RAs were local residents, with undergraduate degrees, who had experience as data collectors in previous maternal health research.

Given cultural tradition related to the veil system, women are not allowed to participate in public meetings in the presence of men. To respect the cultural values and participants’ preferences, FGs were held separately with women and men. Likewise, separate FGs were organized for LHWs, MDs, and health decision/policy makers given the logistics and ease of care providers. Data saturation was determined through a review of FG transcripts for new emerging codes/ideas, and the saturation point was deemed to have been reached when transcripts returned no new codes.

### Data Analysis and Quality Control

FG discussions were taped using a digital voice-recorder and transcribed verbatim in Sindhi. The transcripts were imported to NVivo version 10 software (QSR, Doncaster Vic, Australia) for data analysis. The analysis of FG data was conducted in the same language (Sindhi), using the new version of NViVo that allows for coding in languages other than English. This increased rigor in the data analysis and prevented meaning loss from translation. The participants’ attributes were analyzed, and their responses were coded on a hierarchy of tree nodes (i.e., branches of relevant constructs). A combined approach to data analysis inclusive of inductive and deductive reasoning was used to interpret emerging themes/sub-themes (19). The descriptive coding list provided a comprehensive understanding of thematic areas for subsequent interpretations (Table 2).

**Table 2.** Descriptive Coding List for Thematic Analysis

	Thematic areas related to cost drivers using societal perspective		
Program implementation	OOP costs	Health system costs	Program implementation costs
Patient screening, and out-patient visits	Diagnostic procedures	Human resource [personnel time]	Procurement of technologies for patient screening
	Out-patient doctor's visit	Laboratory tests	Trainings for technology-use
			mHealth platform to support technology implementation
Referral and transport to health facility	Transport to-and-from health facility	Transport for home-based visits	Cellular communication
	Missed wages by family and/or care-givers	Accompanying women to hospital	Access to computer and internet
Treatment	In-patient hospitalization, other than childbirth	Initial case-management for sick women	Monitoring and feedback
	In-patient hospitalization for childbirth	Clinical procedures
	Medications	Medications
	Care received from traditional care-providers	Specialist consultations
Community engagement	Opportunity costs to attend sessions	Personnel time	Materials, and logistic support

Data quality was ensured through random observations of FGs by field coordinators and a public health scientist, 10 percent (audit-trail) verifications of the content of manual transcripts by audio-recording reviews, and weekly debriefing sessions with moderators/transcribers. The FG moderator recorded a self-reflection after each session to describe personal thoughts and impressions to better contextualize the data, as well as to protect against self-bias.

### Ethical Considerations

Written consent was obtained from every participant before conducting the study. This study received ethical approval from the Ethics Review Committee (ERC) of the Aga Khan University, Karachi, Pakistan (ERC # 3230-OBS-ERC-14), and the Institutional Review Board of the University of British Columbia in Vancouver, Canada (ETHICS # H12-00132), as the central coordinating site.

## RESULTS

Participants’ discussions were categorized into emerging themes and sub-themes as HDP-related health resource usage, and/or as perceived costs to family, health system, and program implementation.

### Healthcare Resource Usage for HDP

The theme on resource usage suggested patterns and types of care sought from health facilities in the catchments. Almost all pregnant women reported having sought health care after they were identified as hypertensive and referred to a health facility by LHWs. The majority visited public secondary and tertiary health facilities that were usually located outside study clusters, requiring families to arrange transport. In most cases, women were delivered by Cesarean delivery (C-section). One woman described her experience of visiting a distantly located public health facility in the following quote:

“We traveled to Shadadpur government hospital for the treatment. Doctors told . . . [me] . . . that my blood pressure was extremely high and I had to deliver the baby. They said that normal delivery is unlikely because of high blood pressure, therefore, they did C-section to deliver my baby”.

Participant 6, FG 1, pregnant women and mothers-in-law

A few pregnant women stated that they visited private general practitioners because of easy access, family preferences, and past unfavorable experiences with public sector health facilities.

### Perceived Cost Drivers

The theme on perceived cost drivers contextualized a broad range of costs to care receivers, healthcare providers, and decision/policy makers.

#### OOP Costs

Pregnant women (i.e., care-receivers) emphasized OOP expenditure as the main barrier to seeking timely care. The OOP costs were described as in- and out-patient care, diagnostic procedures, informal care received from traditional healers, self-medication at home, childbirth, and transport to health facilities. The reported lump sum OOP costs ranged from 50 Pakistani rupees (PKR) (USD0.5) for diagnostic procedures to 10,000 PKR (USD100) for in-patient hospitalization for childbirth (Table 3).

**Table 3.** Lump Sum OOP Costs Related to Healthcare Sought for HDP

	Cost in PKR (USD)	
Health resource utilization	Minimum	Maximum
Diagnostic procedures	50 (0.5)	3500 (35.0)
Out-patient doctor's visit	150 (1.5)	1500 (15.0)
In-patient hospitalization, other than childbirth	2500 (25.0)	7000 (70.0)
In-patient hospitalization for childbirth	3000 (30.0)	10000 (100.0)
Transport to-and-from health facility	500 (5.0)	3500 (35.0)
Childbirth at home [Conducted by traditional birth attendant]	1000 (10.0)	1500 (15.0)
Self-medication	800 (8.0)	2000 (20.0)
Missed wages (inclusive of woman and caregiver)	900 (9.0)	5000 (50.0)

USD currency exchange rate of 2015.

The OOP costs were usually perceived as a large financial burden for the family of a pregnant woman, and they had a substantial impact on their economic conditions and wellbeing. One husband described this in the following quote:

“We don't have savings for emergencies related pregnancy and childbirth. [We had to sell] family assets and borrowed money from friends/neighbors. . . It took us several months to pay back the borrowings.”

Participant 7, FG 1, husbands and fathers-in-law

Alternatively, costs deterred families from seeking care:

“Because I did not have money, we didn't go to hospital when my wife was identified as hypertensive during the current pregnancy. . . .we cannot afford health care expenses!”.

Participant 7, FG 2, husbands and fathers-in-law

Participants stated that husbands (in most cases) and/or mothers-in-law were responsible for accompanying pregnant women when they were transported to a health facility. Additionally, blood relatives, and/or family friends were often reported to have stayed with, and remained involved in patient care, when a pregnant woman was hospitalized for HDP management and for childbirth. A vast majority of family members were paid a lump sum wage of 150 to 500 PKR (USD 1.5 to 5) per day, or a fixed salary of 8,000 to 13,000 PKR (USD 80 to 130) per month. Consequently, missed wages for family members were considered to be a double economic burden for the family and others involved.

#### Health System Costs

LHWs and MDs (i.e., care providers) revealed health system costs associated with human resources, transport, and communication. LHWs described making once-monthly home visits to provide basic antenatal care to pregnant women in their catchment areas. Home visits were described to include screening pregnant woman for HDP, initial case management (if the pregnant woman was identified at risk for adverse birth outcomes), and referrals to health facilities. The duration of a completely routine visit, where the pregnant woman was found to be in good health, was 20 to 30 minutes. A more typical visit would take 30–60 minutes to complete if the pregnant woman required medications and/or the family needed counseling about referral to a health facility. One LHW described her typical home visit experience in the following quote:

“A pregnant woman would often speak about her problems. Most women have a problem of anemia and complain of pain. They share domestic worries related to their husband's lack of support and misunderstandings with mothers-in-law. Therefore, it sometimes takes one hour to complete a visit.”

Participant 6, FG 1, LHWs

Moreover, a few LHWs mentioned families’ expectations that they (LHWs) accompany pregnant women to healthcare facilities and exchange their cell phone numbers to maintain communication for emergencies.

MDs practicing at primary and secondary health facilities considered performing basic diagnostic tests such as blood pressure monitoring, blood sugar testing, proteinuria measurement, and ultrasound imaging. Prescribing patterns revealed frequent use of oral tablet methyldopa (Aldomet) to treat high blood pressure, injection [of] diazepam to control seizures, and referral to higher-level health facility for severe complications during pregnancy. MDs practicing at tertiary-level health facilities considered advanced maternal and fetal tests, such as 24-hour urine collection, blood tests (i.e., serum uric acid, prothrombin time, activated partial thromboplastin time, electrolyte profile, complete blood count, lipid profile, liver function tests), and fetal imaging. All recommended close monitoring as an in-patient and interventions that included intravenous administration of magnesium sulfate, blood transfusion, and Cesarean delivery.

#### Program Implementation Costs

Health decision/policy makers recognized program implementation costs (such as mobile health infrastructure, staff training, and monitoring/supervision) as major investments for the health system. All health decision/policy makers believed that mHealth technologies have potential to improve maternal and perinatal health in LMICs. They discussed the prerequisites for effective program implementation: (i) a major initial investment in establishing the mHealth platform (e.g., purchase of electronic tablet devices for app-guided clinical care, computers for downloading and data-sharing, and high speed Internet to facilitate data synchronization), and (ii) patient screening equipment (e.g., digital blood pressure devices, pulse oximeters, and urine dipsticks). Many health decision/policy makers reported the need to train all existing staff through refresher programs and to provide ongoing monitoring and supervision.

## DISCUSSION

This study reports a societal perspective of cost drivers relevant to stakeholders considering the CLIP interventions, and has demonstrated that care-seeking practices vary between public and private health sectors in Sindh, Pakistan. Our findings further highlighted contextual aspects of resource usage that guided the design of a comprehensive questionnaire for quantitative ascertainment of individual level health resource usage during the trial period.

Given ever-increasing reliance on technology use in health care, there are implications for incremental costs to society (20). We found that referral of pregnant women requiring treatment at health facility resulted in the OOP costs, and productivity loss/missed wages further added financial burden on families and/or primary caregivers. Our findings are corroborated with other studies from developing countries that indicate over 50 percent of total health spending as OOP (21). Ironically, OOP costs are often omitted from cost analyses because such costs are assumed to be irrelevant for program implementation. A recent cost analysis of peer health workers and mHealth support interventions for improving AIDS care in Uganda lacked OOP costs in their analysis (22). We also interpreted from FGs that the sicker a patient was, in terms of severity and complication, and/or if the decisions to seek care were delayed, the higher the OOP costs incurred. Thus, our findings point to the need to consider OOP costs when conducting economic evaluation of health technologies in LMICs.

The program implementation as indicated in this study suggests incremental costs to the provincial public health department, as health technology procurement and trainings require substantial investments. Program costs are often feared to increase the burden on existing health budgets in LMICs. It is likely that community-based screening and/or interventions using mHealth platform will influence care-seeking behaviors. Our argument is supported by an RCT that demonstrated that short message services (SMS) and cell phone reminders (i.e., the intervention group), compared with no reminders (i.e., the control group), were associated with significantly higher attendance rates at health promotion centers (23). Another cost-effectiveness study from Pakistan reported higher costs related to personnel time, equipment and supplies in community-based response stimulation and nutrition interventions on early child development (24).

The economic appraisal of emerging health technologies plays a pivotal role in policy advocacy and resource allocation for posttrial program scale-up (11). We found that health decision/policy makers supported mHealth initiative, and expressed their interest in finding cost-effectiveness of the CLIP interventions in Sindh, Pakistan. These findings are similar to those observed in a qualitative study in the United Kingdom, where health authorities strongly recommended economic evaluation to advise policy decisions in health care (25).

### Strengths and Limitations

The International Society of Pharmacoeconomics and Outcome Research (ISPOR) recommend in-depth assessment of jurisdiction-specific costs and outcome parameters for decision-analytical models of multi-national trials. In this study, we used a pragmatic approach to assess cost drivers necessary for the economic analysis alongside the CLIP trial in Sindh, Pakistan. FGs with a wide range of stakeholders in relation to the CLIP trial further add methodological rigor to the overall study. We recognize that combine FGs for pregnant women- and mothers-in-law; husbands- and fathers-in-law may have resulted in social desirability bias and it is the main limitation of current study.

The transferability of our study findings is limited to care-receivers, care-providers, and program implementers in relation to the CLIP trial in Sindh, Pakistan. The methodological approaches as reported in this study may guide future health economics studies evaluating MNCH interventions in Pakistan and other LMICs.

## CONCLUSIONS

A thorough understanding of care-seeking practices revealed financial implications for families of pregnant women, and program costs for the health system during implementation of CLIP trial. The societal perspective provided contextual information, and revealed a more comprehensive description of cost drivers that can be used to design an economic model to fulfill the needs of health decision/policy makers considering CLIP interventions in Sindh, Pakistan.

## CONFLICTS OF INTEREST

The author(s) declare that they have no competing interests.
